# Reducing power and iron chelating property of *Terminalia chebula* (Retz.) alleviates iron induced liver toxicity in mice

**DOI:** 10.1186/1472-6882-12-144

**Published:** 2012-08-31

**Authors:** Rhitajit Sarkar, Bibhabasu Hazra, Nripendranath Mandal

**Affiliations:** 1 Division of Molecular Medicine, Bose Institute, P-1/12 CIT Scheme VIIM, Kolkata 700054, India

## Abstract

**Background:**

The 70% methanol extract of *Terminalia chebula* Retz. fruit (TCME) was investigated for its *in vitro* iron chelating property and *in vivo* ameliorating effect on hepatic injury of iron overloaded mice.

**Methods:**

The effect of fruit extract on Fe^2+^-ferrozine complex formation and Fe^2+^ mediated pUC-18 DNA breakdown was studied in order to find the *in vitro* iron chelating activity. Thirty-six Swiss Albino mice were divided into six groups of: blank, patient control and treated with 50, 100, 200 mg/kg b.w. of TCME and desirox (standard iron chelator drug with Deferasirox as parent compound). Evaluations were made for serum markers of hepatic damage, antioxidant enzyme, lipid per oxidation and liver fibrosis levels. The reductive release of ferritin iron by the extract was further studied.

**Results:**

*In vitro* results showed considerable iron chelation with IC_50_ of 27.19 *±* 2.80 μg/ml, and a significant DNA protection with [P]_50_ of 1.07 ± 0.03 μg/ml along with about 86% retention of supercoiled DNA. Iron-dextran injection (i.p.) caused significant increase in the levels of the serum enzymes, viz., alanine aminotransferase (ALAT), aspartate aminotransferase (ASAT), alkaline phosphatase (ALP) and Bilirubin, which were subsequently lowered by oral administration of 200 mg/kg b.w. dose of the fruit extract by 81.5%, 105.88%, 188.08% and 128.31%, respectively. Similarly, treatment with the same dose of the extract was shown to alleviate the reduced levels of liver antioxidant enzyme superoxide dismutase, catalase, glutathione S-transferase and non-enzymatic reduced glutathione, by 49.8%, 53.5%, 35.4% and 11% respectively, in comparison to the iron overloaded mice. At the same time, the fruit extract effectively lowered the iron-overload induced raised levels of lipid per oxidation, protein carbonyl, hydroxyproline and liver iron by 49%, 67%, 67% and 26%, respectively, with oral treatment of 200 mg/kg b.w. dose of TCME. The fruit extract also showed potential activity for reductive release of ferritin iron.

**Conclusions:**

These findings suggest that *Terminalia chebula* extract may contain active substances capable of lessening iron overload induced toxicity, and hence possibly be useful as iron chelating drug for iron overload diseases.

## Background

Iron is an important trace element of the body, being found in functional form in hemoglobin, myoglobin, the cytochromes, enzymes with iron sulphur complexes and other iron-dependent enzymes [1]. Iron has the unique ability to alter its oxidation and redox states in response to liganding, which makes it essential for various cellular processes [2]. The cells maintain the free iron concentration to a minimum required level to avoid toxic effects of excess iron. But, in some situation the iron balance is disrupted and resulting in iron overload which is associated with the oxidative stress induced several health problems including anemias, heart failure, liver cirrhosis, fibrosis, gallbladder disorders, diabetes, arthritis, depression, impotence, infertility, and cancer [3]. The body lacks any effective means to excrete excessive iron and therefore the interest has been grown to develop the potent chelating agent capable of complexing with iron and promoting its excretion [4,5].

*Terminalia chebula* Retz. (abbreviated as TC) from Combretaceae family is an important medicinal herb which grows throughout central Asia and some other parts of the world [6]. The dried ripe fruit of TC is used widely in the indigenous system of medicine (ayurvedic) for its homeostatic, antitussive, laxative, diuretic, and cardiotonic activities [7] and serves as a major component of widely used ayurvedic formulation, ‘Triphala’ [8]. TC extract has also been reported to exhibit a variety of biological activities including antioxidant [9-11], anticancer [12-14], cytoprotective [15], antidiabetic [16,17], antibacterial [18], gastro protective [19] and hepatoprotective [20] activities. The phytochemical analysis shows that TC is a rich source of various phenolic and flavonoid compounds [21] which are well known for their free radical scavenging and iron chelation property [22]. Earlier, we have also reported the ROS scavenging and reducing property of 70% methanol extract of *T. chebula* and it also found to possess significant amount of phenolic and flavonoid compounds [23].

Iron overload increases the formation of reactive oxygen species (ROS) which involves the initiation of lipid peroxidation, protein oxidation and liver fibrosis. However, excess iron is stored as Fe^3+^ in ferritin and iron overload sustains for long period if the stored iron is not getting reduced and released because the efficiency of iron chelating drugs depend on the reductive release of ferritin iron [24]. Moreover, 70% methanol extract of *T. chebula* was earlier reported to contain some notable antioxidants, viz., ellagic acid, 2,4-chebulyl-β-D-glucopyranose and chebulinic acid [12]. Based on these observations, the present study was performed to assess iron chelating activity of 70% methanol extract of *T. chebula* (TCME) and whether this activity along with reducing power can normalize the damage caused to liver by iron overload.

## Methods

### Chemicals

Iron-dextran and guanidine hydrochloride was purchased from Sigma-Aldrich, USA. Trichloroacetic acid (TCA), nitro blue tetrazolium (NBT), reduced nicotinamide adenine dinucleotide (NADH), phenazine methosulfate (PMS), ferrozine, glutathione reduced, bathophenanthroline sulfonate disodium salt, thiobarbituric acid (TBA), and 5,5′-dithiobis-2-nitrobenzoic acid (DTNB) were obtained from Sisco Research Laboratories Pvt. Ltd, Mumbai, India. Hydrogen peroxide, ammonium iron (II) sulfate hexahydrate [(NH_4_)_2_Fe(SO_4_)_2_6H_2_O], 1-chloro-2,4-dinitrobenzene (CDNB), chloramine-T, hydroxylamine hydrochloride, dimethyl-4-aminobenzaldehyde and 2,4-dinitro phenylhydrazin (DNPH) were obtained from Merck, Mumbai, India. Ferritin was purchased from MP Biomedicals, USA. Streptomycin sulphate was obtained from HiMedia Laboratories Pvt. Ltd, Mumbai, India. The standard oral iron chelating drug, desirox, with the parent group Deferasirox, was obtained from Cipla Ltd., Kolkata, India.

### Plant material

The fruits of TC were collected from Bankura district of West Bengal, India. It was identified and authenticated by the Central Research Institute (Ayurveda), Kolkata, India and a voucher specimen (CRHS 113/08) was submitted there.

### Animals

Male Swiss albino mice (20 *±* 2 g) were purchased from Chittaranjan National Cancer Institute (CNCI), Kolkata, India and were maintained under a constant 12 h dark/light cycle at an environmental temperature of 22 ± 2°C. The animals were provided with normal laboratory pellet diet and water *ad libitum*. All experiments were performed after obtaining approval from the Institutional Animal Ethics Committee, with certified regulations of the Committee for the Purpose of Control and Supervision of Experiments on Animals (CPCSEA), Ministry of Environment and Forest, Govt. of India (Bose Institute Registration. No. 95/1999/CPCSEA).

### Preparation of plant extract

The powder (100 g) of the air dried fruits of TC was stirred using a magnetic stirrer with 500 ml mixture of methanol: water (7:3) for 15 h; then the mixture was centrifuged at 2850 x *g* and the supernatant decanted. The process was repeated again with the precipitated pellet. The supernatants were collected, concentrated in a rotary evaporator and lyophilized. The dried extract, denoted as TCME was stored at −20*°*C until use. An aqueous solution with various concentrations of TCME was used for all the experiments.

### In vitro study

#### Iron chelation

The chelating activity of TCME for ferrous ion was evaluated by a previously described method [25]. In a Hepes buffer (20 mM, pH 7.2) medium, TCME (0–120 μg/ml) was added to ferrous sulfate solution (12.5 μM) and the reaction was started by the addition of ferrozine (75 μM). The mixture was shaken vigorously and left standing for 20 min at room temperature. The absorbance was then taken at 562 nm. All tests were performed six times. EDTA was used as a positive control.

#### DNA protection

The DNA protection was studied using supercoiled pUC18 plasmid DNA according to an earlier reported method [26], with minor modifications. In Hepes buffer (pH 7.2, 100 mM), FeSO_4_ solution (750 μM), TCME of varying doses (0–5 μg/ml), DNA (0.5 mg/ml) and water were added to make an initial reaction mixture. Finally, H_2_O_2_ solution (7.5 mM) was added to start the reaction. After 10 min, the reaction was terminated by adding Desferal as stopping reagent followed by loading buffer. 25 μl of each reaction mixture was loaded in 1% agarose gel. After migration, the gel was stained with ethidium bromide and visualized in a UV transilluminator. The DNA bands were quantified through densitometry and the following formulae were used to calculate the percentage of protection.

$$\%SC=\left[1.4\times SC/\left(OC+\left(1.4\times SC\right)\right)\right]\times 100$$

where, SC = supercoiled; OC = open circular; 1.4 = correction factor

$$\%protection=100\times \left[\left(controlSC-chelatorSC\right)/\left(controlSC-nochelatorSC\right)-1\right]$$

The ability of the fruit extract to protect the DNA supercoil can be expressed by the concentration of sample required for 50% protection, designated as the [P]_50_ value.

### In vivo study

#### Experimental design

Thirty-six mice were distributed into six groups comprising six mice in each group. One group received normal saline only and served as blank (B). The other five groups were given five doses (one dose every two days) of 100 mg/kg b.w. each, of iron-dextran saline (i.p). Normal saline was administered to one iron-dextran group (C) and other four groups were orally treated with 50 mg/kg b.w. (S50), 100 mg/kg b.w. (S100), 200 mg/kg b.w. (S200) TCME and 20 mg/kg b.w. desirox (D), respectively, for three consecutive 7 day periods, started from the day after the first iron-dextran injection.

#### Sample collection and tissue preparation

Mice were fasted overnight after the experiment ended on the 21^st^ day. Then they were anesthetized by ethyl ether and blood was collected by cardiac puncture. After the clotting of blood samples, sera were separated by centrifugation and stored at −80°C until analysis. The liver was dissected out and blood cells were eliminated after rinsing with ice-cold saline, half of them were cut, weighed and homogenized in 10 volume of 0.1 M phosphate buffer (pH 7.4) containing 5 mM EDTA and 0.15 M NaCl, and centrifuged at 8000 x *g* for 30 min at 4°C. The supernatant was collected and used for the determination of lipid per oxidation, protein oxidation, hydroxyproline content and enzyme activities. A standard graph of BSA was prepared to estimate the protein concentration in the homogenate by Lowry method [27]. The other half of the liver samples were weighed and digested with equivolume (1:1) mixture of sulphuric acid and nitric acid and their iron content were analysed.

#### Serum enzymes

Alanine aminotransferase (ALAT), aspartate aminotransferase (ASAT) and bilirubin in serum samples were measured using the commercial kits of Merck, Mumbai, India. Alkaline phosphatase (ALP) was estimated using the kit supplied by Sentinel Diagnostics, Italy.

#### Antioxidant enzymes

Superoxide dismutase (SOD) was assayed by measuring the inhibition of the formation of blue colored formazan at 560 nm according to the technique reported previously [28]. Catalase (CAT) activity was measured by following the decomposition of H_2_O_2_ over time at 240 nm according to a previously described method [29]. Glutathione-S-transferase (GST) was determined by a formerly reported method [30] based on the formation of GSH-CDNB conjugate and increase in the absorbance at 340 nm. Reduced glutathione (GSH) level was measured spectrophotometrically at 412 nm by a standard method [31].

#### Lipid peroxidation products

According to a formerly reported method [32], the lipid peroxide levels in liver homogenates were measured in terms of thiobarbituric acid reactive substances (TBARS), as an index of malondialdehyde accumulation.

#### Protein carbonyl content

As a marker of protein oxidation, protein carbonyl contents were estimated spectrophotometrically by a previously described method [33]. Briefly, 450 μl sample homogenate was mixed with 50 μl streptomycin sulphate (10% w/v) and then centrifuged at 2800 g for 15 min. Then 200 μl of the supernatant was incubated with the same volume of 10 mM DNPH in 2 M HCl at room temperature for 20 min. After the reaction was completed, 10% cold TCA was added to precipitate the proteins and the precipitates were washed with ethyl acetate-ethanol mixture (1:1) for three times to remove unreacted DNPH. The final protein pellet was dissolved in 1 ml of 6 M guanidine hydrochloride solution and the absorbance was measured at 370 nm, using the molar extinction coefficient of DNPH, ε = 2.2x10^-4^ M^-1^ cm^-1^.

#### Hydroxyproline content

Hydroxyproline content represents the content of collagen, which is closely related with liver fibrosis. Liver samples were hydrolized in 6 M HCl and hydroxyproline was measured by Ehrlich’s solution according to the method described previously [34]. A standard curve (*R*^*2*^ = 0.9907) of 4-hydroxy-L-proline was prepared and results were calculated after taking absorbances at 558 nm. The collagen content was determined by multiplying amount of total hydroxyproline content in each sample by a factor of 7.69 [35]. Results are expressed as milligrams of collagen per liver (wet weight).

#### Liver iron and serum ferritin

Liver iron was measured according to a formerly reported colorimetric method [36]. Samples were incubated with bathophenanthroline sulfonate for 30 min at 37 °C and absorbances were measured at 535 nm. Serum ferritin levels were measured using enzyme-linked immunosorbent assay kit (from Monobind Inc., USA) according to the manufacturer’s instructions.

#### Iron release from ferritin

Iron release assay was performed according to a previously described method [37]. The release of ferritin iron was measured using the ferrous chelator ferrozine as a chromophore. The reaction mixture (3 ml final volume) contained 200 μg ferritin, 500 μM ferrozine, in 50 mM pH 7.0 phosphate buffer. Reaction was started by the addition of 500 μl TCME of different concentrations (100–500 μg) and the change in absorbance was measured continuously at 560 nm for 20 min. A cuvette containing ferritin, ferrozine and phosphate buffer but lacking plant extract was used as the reference solution.

### Statistical analysis

All data are reported as the mean ± SD of six measurements. Statistical analysis was performed using KyPlot version 2.0 beta 15 (32 bit) and Origin professional 6.0. Comparisons among groups were made according to pair *t*-test. The IC_50_ values were calculated by the formula, Y = 100*A1/(X + A1) where A1 = IC_50_, Y = response (Y = 100% when X = 0), X = inhibitory concentration. In all analyses, a *p* value of < 0.05 was considered significant.

## Results

### *In vitro* study

**Figure 1:**
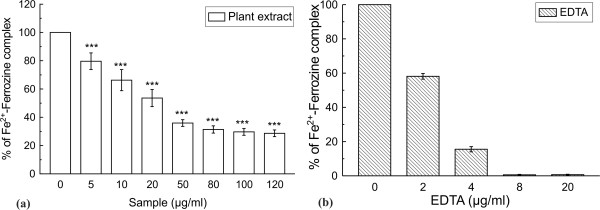
**In vitro *iron chelating activity *.** Effect of (**a**) TCME and (**b**) standard EDTA on ferrozine-Fe^2+^ complex formation. The data expressed as % inhibition of chromogen formation. The results are mean ± S.D. of six parallel measurements. ***p < 0.001 vs 0 μg/ml.

Ferrozine makes a violet colored complex with Fe^2+^ ion. The complex formation is interrupted in presence of chelating agent and as a result the violet color of the complex is decreased. The results [Figures 1(a) and 1(b)] demonstrated that the formation of ferrozine-Fe^2+^ complex is inhibited in the presence of TCME and reference compound EDTA. The IC_50_ values of the TCME and EDTA were 27.19 *±* 2.80 μg/ml and 1.27 *±* 0.05 μg/ml, respectively. At 120 μg/ml, 69% of the percentage of inhibition of complex formation by the TCME was found.

**Figure 2:**
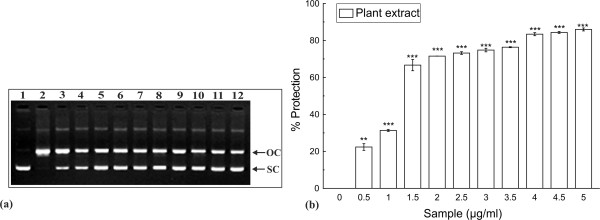
**Inhibition of DNA damage*.*** Protection against oxidative damage to pUC18 by TCME. Picture of agarose gel of pUC18 DNA showing bands of supercoiled (SC) and open circular (OC) forms. Lanes on the gel represent: (Lane 1) control DNA (no H_2_O_2_ or Fe^2+^); (Lane 2) reaction mixture without extract; (Lane 3–12) reaction mixture with extract of increasing concentration (0.5-5 μg/ml). **p < 0.01 and ***p < 0.001 vs 0 μg/ml.

The protective effect of TCME against Fe^2+^-H_2_O_2_ mediated DNA breakdown was demonstrated in Figure 2(a). pUC18 supercoiled DNA was used as control (lane 1). The treatment of supercoiled DNA with Fenton’s reagent led to the conversion of DNA to open circular form (lane 2). The addition of different concentrations of TCME resulted in the restoration of DNA in the supercoiled form (lane 3–12). The results in Figure 2(b) showed the dose dependant protective effect of TCME with a [P]_50_ value of 1.07 ± 0.03 μg/ml.

### *In vivo* study

#### Serum enzymes

Iron induced liver injury resulted in the significant increase in the levels of ALAT, ASAT, ALP and bilirubin. As shown in Table 1, oral administration of TCME in S200 group markedly reduced the levels of ALAT by 81.5%, ASAT by 105.88%, ALP by188.08%; and bilirubin by 128.31%, whereas a reduction of 74.29%, 83.55%, 185.04% and 124.87% was found in the standard desirox treated group for ALAT, ASAT, ALP and bilirubin, respectively.

**Table 1:** The effect of TCME on serum marker enzymes (ALAT, ASAT and ALP) and Bilirubin in iron overloaded mice

**Treatment**	**ALAT**		**ASAT**		**ALP**		**Bilirubin**	
	**Unit/L**	**% Change**	**Unit/L**	**% Change**	**Unit/L**	**% Change**	**Unit/L**	**% Change**
**B**	16.87 ± 2.07	---	30.07 ± 1.77	---	133.58 ± 7.76	---	1.29 ± 0.15	---
**C**	36.08 ± 2.76^X2^	113.79	69.8 ± 1.46^X3^	132.13	398.76 ± 24.73^X2^	198.51	3.21 ± 0.23^X3^	147.88
**S50**	33.27 ± 2.91^X2^	97.12	50.67 ± 3.58^X2Y2^	68.51	265.11 ± 19.09^X2Y3^	98.45	2.45 ± 0.19^X3Y2^	88.89
**S100**	30.85 ± 2.53^X2^	82.75	43.71 ± 1.33^X2Y3^	45.35	236.08 ± 12.25^X2Y2^	76.73	1.92 ± 0.19^X2Y3^	48.41
**S200**	22.33 ± 1.99^X1Y1^	32.29	37.96 ± 2.13^X2Y3^	26.25	147.52 ± 12.11^Y2^	10.43	1.55 ± 0.06^X1Y2^	19.57
**D**	23.55 ± 1.39^X1Y1^	39.54	44.67 ± 5.52^X1Y1^	48.58	151.58 ± 9.92^X2Y2^	13.47	1.59 ± 0.12^X2Y2^	23.01

Values are mean ± SD of six observations.

X: significant difference from normal mice (B) group (X1: *p* ≤ 0.05, X2: *p* ≤ 0.01 and X3: *p* ≤ 0.001).

Y: significant difference from iron overloaded (C) group (Y1: *p* ≤ 0.05, Y2: *p* ≤ 0.01 and Y3: *p* ≤ 0.001).

#### Antioxidant enzymes

**Figure 3:**
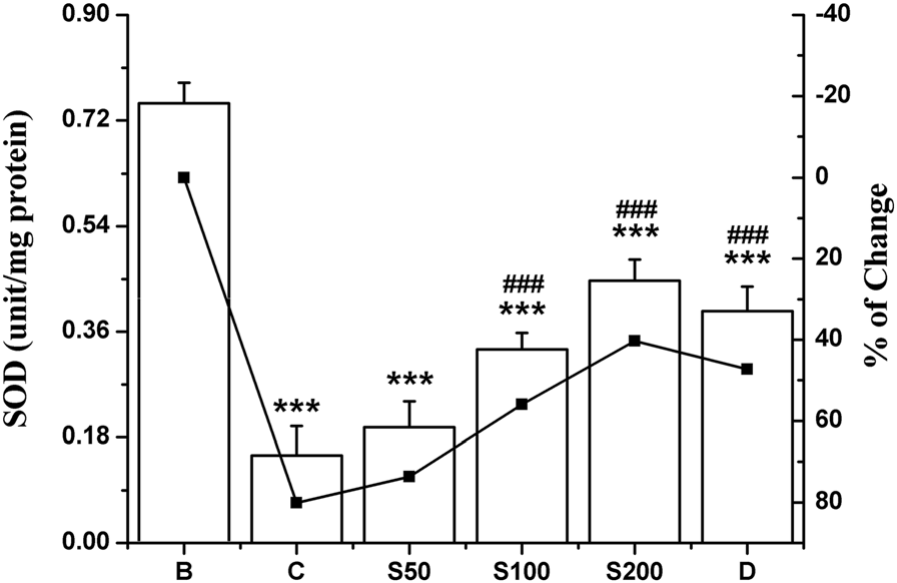
**Increase in SOD level.** Effect of TCME on the SOD levels against iron overload induced hepatic injury in mice. Mice were randomly divided into six groups (blank, B; control, C; 50 mg/kg b.w. TCME, S50; 100 mg/kg b.w. TCME, S100; 200 mg/kg b.w. TCME, S200; desirox group, D) and treated as described in ‘experimental design’ section. Values are expressed as mean ± SD of six mice. ***p ≤ 0.001 compared with blank and ^###^*p* ≤ 0.001 compared with control.

**Figure 4:**
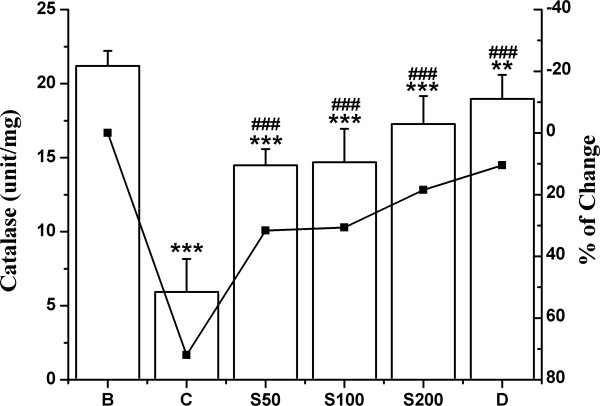
**Elevation of Catalase activity.** Effect of TCME on the CAT levels in iron overload induced hepatic damage in mice. Mice were randomly divided into six groups (blank, B; control, C; 50 mg/kg b.w. TCME, S50; 100 mg/kg b.w. TCME, S100; 200 mg/kg b.w. TCME, S200; desirox group, D) and treated as described in ‘experimental design’ section. Values are expressed as mean ± SD (n = 6). **p ≤ 0.01, ***p ≤ 0.001 compared with blank and ^###^*p* ≤ 0.001 compared with control.

**Figure 5:**
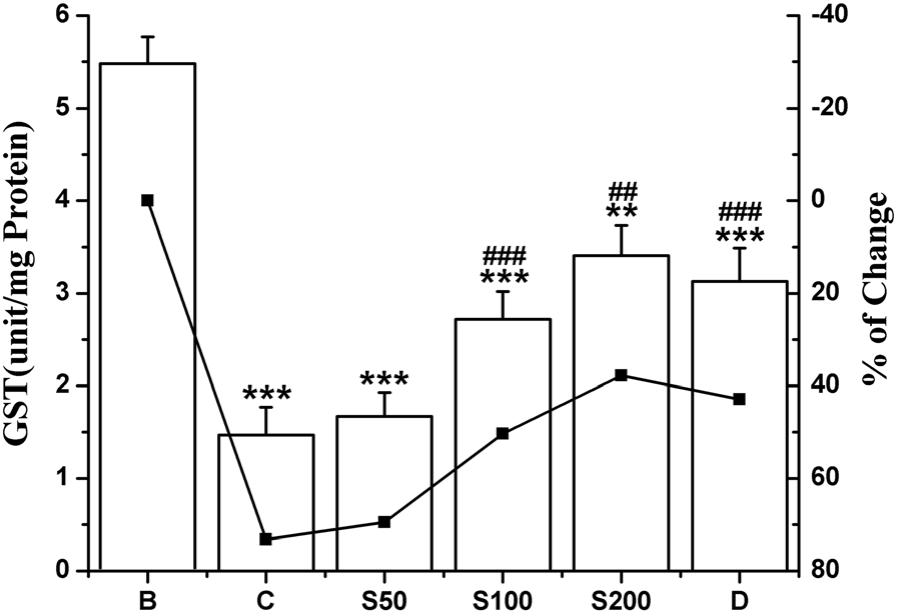
**Effect on GST level.** Effect of TCME on the GST levels against iron overload induced hepatic injury in mice. Mice were randomly divided into six groups (blank, B; control, C; 50 mg/kg b.w. TCME, S50; 100 mg/kg b.w. TCME, S100; 200 mg/kg b.w. TCME, S200; desirox group, D) and treated as described in ‘experimental design’ section. Values are expressed as mean ± SD of six mice. **p ≤ 0.01, ***p ≤ 0.001 compared with blank and ^##^*p* ≤ 0.01, ^###^*p* ≤ 0.001 compared with control.

**Figure 6:**
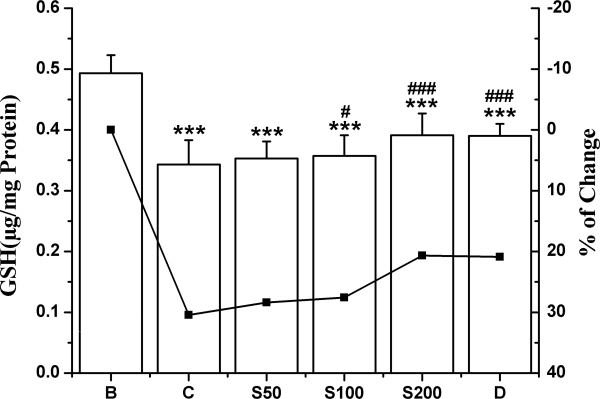
**Increment of GSH activity.** Effect of TCME on the GSH levels against iron overload induced hepatic damage in mice. Mice were randomly divided into six groups (blank, B; control, C; 50 mg/kg b.w. TCME, S50; 100 mg/kg b.w. TCME, S100; 200 mg/kg b.w. TCME, S200; desirox group, D) and treated as described in ‘experimental design’ section. Values are expressed as mean ± SD of six mice. ***p ≤ 0.001 compared with blank and ^#^*p* ≤ 0.05, ^###^*p* ≤ 0.001 compared with control.

The activities of antioxidant enzymes SOD, CAT, GST and the levels of non-enzymic antioxidant GSH were significantly decreased in iron overloaded control mice compared to normal mice. In the liver, 80.1% decrease in SOD activity observed in the test control compared with normal group, was successively reduced to 6.4%, 24% and 39.8% following the feeding of S50, S100 and S200 respectively (Figure 3); and to 32.9% upon treatment with standard desirox. The activity of CAT was shown in Figure 4. The overload of iron in liver resulted in 72% decrease in CAT activity compared to non-iron overloaded normal mice. But 40.4%, 41.3% and 53.5% reduction was observed; when the iron intoxicated mice were fed 50, 100 and 200 mg/kg b.w. of TCME respectively, which approached the value for desirox (61.5%). GST activity was reduced 73.2% in iron treated control mice. But after the treatment with 50, 100 and 200 mg/kg b.w. of TCME, the activity was enhanced 3.7%, 22.8% and 35.4% respectively (Figure 5), the last being better than 30.3% reduction achieved by the reference desirox. The activity of GSH was significantly reduced (31%) in iron intoxicated control mice. The GSH levels in S50, S100 and S200 groups increased about 3%, 5% and 11%, respectively (Figure 6), comparable to the activity of desirox (9.6%).

#### Lipid peroxidation

**Figure 7:**
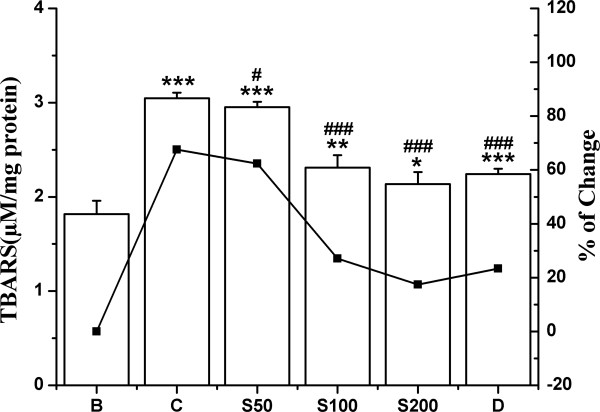
**Inhibition of lipid peroxidation.** Hepatic lipid peroxidation levels in different treated mouse liver. Mice were randomly divided into six groups (blank, B; control, C; 50 mg/kg b.w. TCME, S50; 100 mg/kg b.w. TCME, S100; 200 mg/kg b.w. TCME, S200; desirox group, D) and treated as described in ‘experimental design’ section. Values are expressed as mean ± SD of six mice. *p ≤ 0.05, **p ≤ 0.01, ***p ≤ 0.001 compared with blank and ^#^*p* ≤ 0.05, ^###^*p* ≤ 0.001 compared with control.

The intraperitoneal injection of iron-dextran significantly enhanced (67%) lipid peroxidation in liver homogenates compared to normal control mice. However, the levels of TBARS were markedly reduced to 5%, 40% and 49% in groups S50, S100 and S200 respectively (Figure 7), whereas standard desirox generated 44.2% decrease.

#### Protein carbonyl content

**Figure 8:**
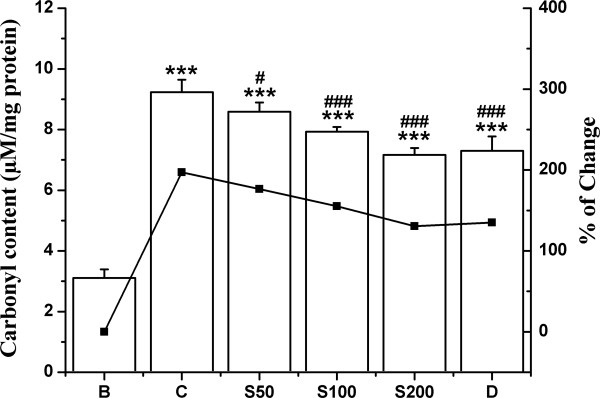
**Effect on protein oxidation.** Inhibitory effect of TCME on protein oxidation levels in iron overloaded mice. Mice were randomly divided into six groups (blank, B; control, C; 50 mg/kg b.w. TCME, S50; 100 mg/kg b.w. TCME, S100; 200 mg/kg b.w. TCME, S200; desirox group, D) and treated as described in ‘experimental design’ section. Protein carbonyl content was assayed to measure the extent of protein oxidation. Values are expressed as mean ± SD (n = 6). ***p ≤ 0.001 compared with blank and ^#^*p* ≤ 0.05, ^###^*p* ≤ 0.001 compared with control.

Oxidative modification of proteins is another consequence of iron overload induced toxicity and carbonyl formation may be an early marker for protein oxidation. A significant elevation (197%) of protein carbonyl content in iron overloaded mice was found to be arrested by 20%, 42% and 67% in S50, S100 and S200 group respectively (Figure 8) as treated with TCME, in comparison to 67% as found for desirox treated group.

#### Hydroxyproline content

**Figure 9:**
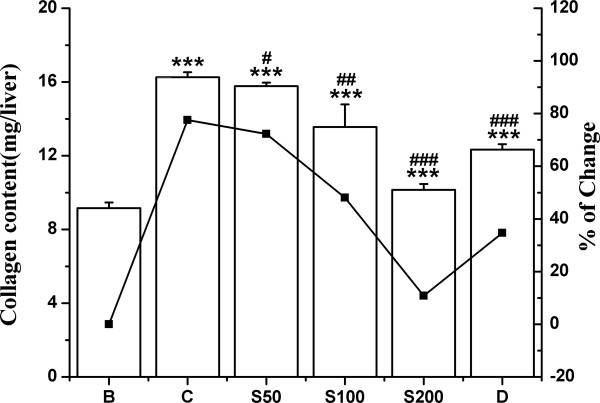
**Effect on collagen content.** Collagen content in different treated mouse liver. Mice were randomly divided into six groups (blank, B; control, C; 50 mg/kg b.w. TCME, S50; 100 mg/kg b.w. TCME, S100; 200 mg/kg b.w. TCME, S200; desirox group, D) and treated as described in ‘experimental design’ section. Values are expressed as mean ± SD (n = 6). ***p ≤ 0.001 compared with blank and ^#^*p* ≤ 0.05, ^##^*p* ≤ 0.01, ^###^*p* ≤ 0.001 compared with control.

The hydroxyproline content was determined as it signifies the enhanced level of collagen content in liver fibrosis. As shown in Figure 9, 77% increase of collagen content in iron overloaded mice compared to normal mice was reduced to 5%, 29% and 67% in TCME treated mice (S50, S100 and S200 respectively); and 43% in D group.

#### Liver iron and serum ferritin

**Figure 10:**
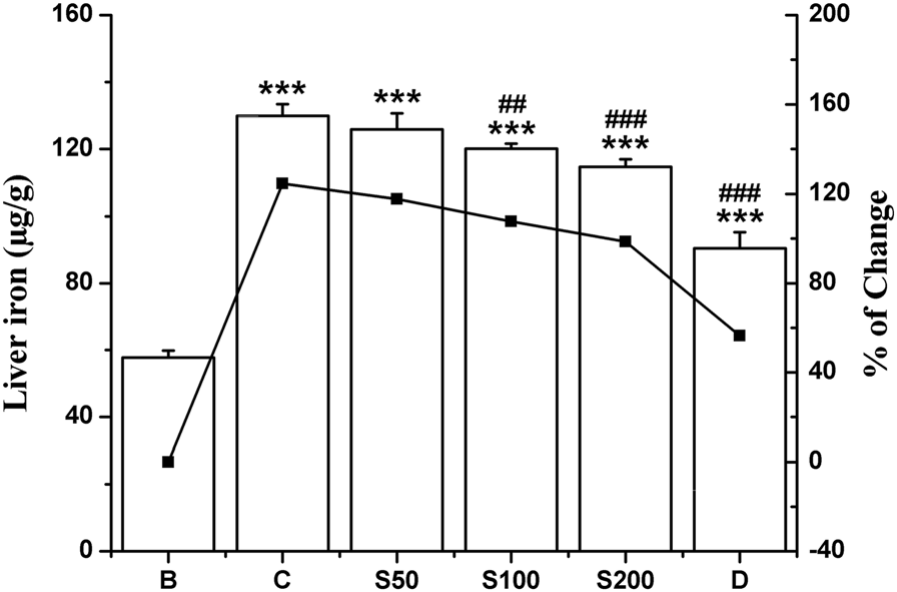
**Hepatic iron content regulation.** Effect of TCME on hepatic iron content in different treated mouse liver. Mice were randomly divided into six groups (blank, B; control, C; 50 mg/kg b.w. TCME, S50; 100 mg/kg b.w. TCME, S100; 200 mg/kg b.w. TCME, S200; desirox group, D) and treated as described in ‘experimental design’ section. Values are expressed as mean ± SD of six mice. ***p ≤ 0.001 compared with blank and ^##^*p* ≤ 0.01, ^###^*p* ≤ 0.001 compared with control.

**Figure 11:**
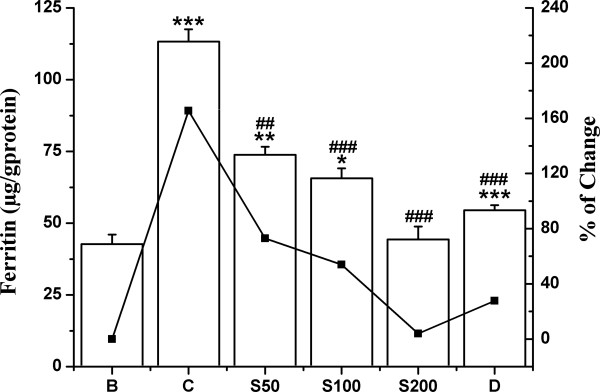
**Decrease of serum ferritin level*.*** Serum ferritin levels in different treated mouse. Mice were randomly divided into six groups (blank, B; control, C; 50 mg/kg b.w. TCME, S50; 100 mg/kg b.w. TCME, S100; 200 mg/kg b.w. TCME, S200; desirox group, D) and treated as described in ‘experimental design’ section. Values are expressed as mean ± SD of six mice. *p ≤ 0.05, **p ≤ 0.01, ***p ≤ 0.001 compared with blank and ^##^*p* ≤ 0.01, ^###^*p* ≤ 0.001 compared with control.

Liver iron content was elevated about 124% after intraperitoneal administration injection of iron dextran. The TCME treatment (S50, S100 and S200) evidently lowered the iron content at a level of 7%, 17% and 26% respectively (Figure 10), although not quite as good as desirox treatment that lowered by 68%. Significant increase (165%) of serum ferritin level in iron loaded mice was substantially reduced to 92%, 111% and 161% as treated with TCME dose dependently to S50, S100 and S200, respectively (Figure 11), the activities being better than that of the desirox group with reduction of 138%.

#### Iron release from ferritin

**Figure 12:**
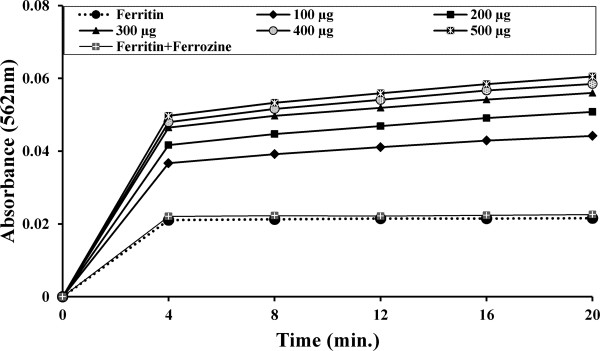
**Release of ferritin iron.** Dose dependent formation of the [Fe(ferrozine)_3_]^2+^ complex following release of Fe^2+^ from ferritin by TCME with time. The reductive release of ferritin iron was quantified by measuring the formation of the ferrous complex of ferrozine, [Fe(ferrozine)_3_]^2+^ at 562 nm using a Shimadzu UV–VIS spectrophotometer.

The ability of TCME to release iron from ferritin was tested using ferrous chelator ferrozine as a chromophore. Ferrozine formed a complex with ferrous ion, [Fe(ferrozine)_3_]^2+^, which was quantified to measure the reductive release of ferritin iron. The time course for the reaction is depicted in Figure 12. Control experiments devoid of TCME produced negligible percent of released iron, whereas, after dose dependant (100–500 μg) addition of TCME, significant amount of iron (45%-63%) was released within 20 minutes.

#### Correlation between reducing power with ferritin iron release

**Figure 13:**
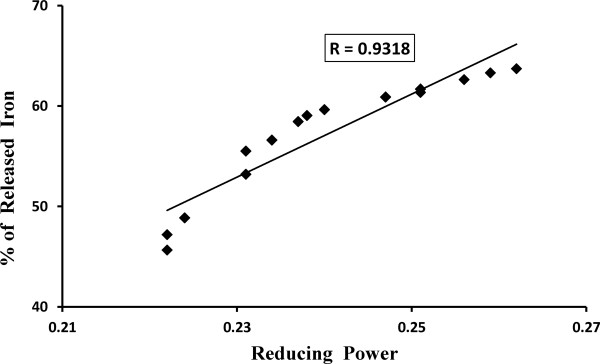
**Correlation of ferritin iron release and reducing power.** Correlation of reducing power versus release of ferritin iron (%) by TCME.

In the present study, the correlation between reducing power and released ferritin iron (%) by TCME was analyzed. The correlation graph is depicted in Figure 13. Results showed a positive correlation coefficient (*R* = 0.9318) between the reducing power and the amount of released ferritin iron (%), which is highly significant (*p* < 0.001).

## Discussion

Iron is the most common cofactor within the oxygen handling biological machinery and, specifically, lipid peroxidation of biological membranes is the main pathogenic mechanism of iron overload induced tissue damage [38]. Harmful effects of extreme iron deposition in liver are likely during iron overload states (e.g., genetic hemochromatosis, thalassemia major and transfusional siderosis). In such conditions, iron has been associated with the initiation and propagation of ROS induced oxidative damage to all biomacromolecules (proteins, lipids, sugar and DNA) that can lead to a critical failure of biological functions and ultimately cell death [39]. An effective therapeutic approach can play a double role in reducing the rate of oxidation - one by sequestering and chelating cellular iron stores [40] and other as radical trap (i.e., antioxidant activity). Since TCME has shown antioxidant and free radical scavenging activity [24], the present study, primarily incorporates the *in vitro* iron chelation potency of TCME, and inhibition of iron mediated DNA breakdown. Consequently, *in vivo* ameliorating effect of TCME on iron accumulation and oxidative damage in liver of iron overloaded mice is studied. Intraperitoneal iron-dextran injection resembled the hemochromatosis secondary to iron loaded anemias (anemias treated with repeated transfusions) and high iron oral intake [41], while avoiding direct interruption of fruit extract on intestinal iron absorption leading to hepatic and serum iron overload.

Intracellular defense mechanism against free radical generation and pathogenesis involves antioxidant enzymes such as SOD, CAT, GST or compounds such as GSH [42]. Excess iron imbalances their levels with excess ROS production thus resulting oxidative stress, followed by peroxidative decomposition of cellular membrane lipids which is a postulated mechanism of hepatocellular injury in iron overload [43]. Alongside, the iron overload generated ROS can lead to oxidation of protein backbone resulting in modification of catalytic and structural integrity of various important proteins [44] contributing to the pathogenesis of liver fibrosis [45]. In turn, hepatic injury by iron results in the leakage of cellular enzymes into the bloodstream, resulting in augmented levels of serum ALAT, ASAT, ALP and bilirubin [1].

The *in vitro* results from Figure 1 suggest that TCME has iron chelating activity, although not as good as the standard EDTA. The significant dose-dependent reduction in the formation of Fe^2+^-dependent hydroxyl radical induced nicked DNA and increase in supercoiled DNA in the presence of TCME reveal its excellent iron chelating activity. The *in vivo* results showed that TCME administration in iron overloaded mice restored the antioxidant enzymes level significantly. Chiefly, the present study demonstrated the lipid peroxidation and protein oxidation inhibiting capability of TCME, which is supposed to be associated with its iron chelating activity. Iron overload causes a significant increase of hydroxyproline, a marker of liver fibrosis. Treatment with TCME significantly reduced hydroxyproline content in iron intoxicated mice, thus demonstrating the hepatic fibrosis inhibitory potency of the fruit extract. Moreover, the direct effect of TCME to reduce hepatic iron content in treated mice supported its iron chelating potency. Above all, TCME reduced the serum enzymes as well as the total Bilirubin levels, indicating its protective effect over liver damage by iron overload and improvement in its functional efficiency.

Ferritin is a ubiquitous intracellular protein that stores iron in a non-toxic ferric form and also helps prevent iron from mediating oxidative damage to cell constituents [46]. Serum ferritin concentration is the most sensitive indicator of the severity of iron overload and its level usually increases when body’s iron stores increase. In this study, the ferritin level was found enhanced in iron overloaded mice, whereas, the level significantly reduced after the treatment with TCME.

Maximum iron chelators depend on the availability of Fe^2+^, which in turn depends on the rate of reductive release of iron from ferritin. Therefore, successive chelation therapy includes the supplementation of ascorbate as reducing agent to increase the availability of storage iron to chelators [47]. Previously, TCME had shown reductive ability [24] as well as in the present study; a significant positive correlation between reducing power and iron released from ferritin has been well established. Therefore, TCME can also be used as drug to treat iron overload as the present results show its reductive release activity of ferritin iron dose dependently as well as time dependently.

## Conclusions

The current investigation of 70% methanolic extract of *Terminalia chebula* showed that the extract which possesses both reducing power and iron chelating activity can reduce the toxic level of iron in iron overloaded mice and hence protect liver from oxidative stress and fibrosis. Taken together, the current findings will be of use in elucidating the pharmacology and application of TCME as a potential iron chelating drug in the treatment of iron overload diseases.

## Competing interests

The author(s) declare that they have no competing interests.

## Authors’ contributions

RS: Performed the study and drafted the manuscript; BH: Designed and performed the study; NM: Supervised the study design along with drafting the manuscript. All authors read and approved the final manuscript.

## Pre-publication history

The pre-publication history for this paper can be accessed here:

http://www.biomedcentral.com/1472-6882/12/144/prepub
